# Supplementary material for: MetaRibo-Seq measures translation in microbiomes
Source: Nat Commun. 2020 Jun 29;11:3268. doi: 10.1038/s41467-020-17081-z (PMC7324362; doi:10.1038/s41467-020-17081-z)
Supplement: Supplementary file 10 — Supplementary Data 7 [file 41467_2020_17081_MOESM10_ESM.zip › File2/Confidence_High_Taxonomy/320584_out.krona.html]

Javascript must be enabled to view this page.

members
magnitude
magnitudeUnassigned
count
unassigned
taxon
rank

320584\_out

2

2
2
superkingdom

976
2
phylum

2
200643
class

2
171549
order

family
2
815

genus
2
816

1

SRS013215\_contig\_number\_66
357276
species

species
1

SRS024549\_contig\_number\_4150
821
